# Clinical Profiles and Outcomes of Prosthesis-Specific Infective Endocarditis Subsequent to Transcatheter Versus Surgical Aortic Valve Replacement: A Systematic Review and Meta-Analysis

**DOI:** 10.7759/cureus.59398

**Published:** 2024-04-30

**Authors:** Cecilia Monaci, Anandita N Nair, Sai Supraja Gilukara, Thanmayee Tummala, Shreenithi J, Sahar Fatima, Riya Gupta, Nagma Sabu, Hira M Nagra, Annel V Colca Herrera, Mohammed Al-Tawil

**Affiliations:** 1 Cardiovascular Disease, University of Turin, Turin, ITA; 2 Medicine, Our Lady of Fatima University, Valenzuela, PHL; 3 Surgery, Mamata Medical College, Khammam, IND; 4 Internal Medicine, Bhaskar Medical College, Hyderabad, IND; 5 Internal Medicine, Stanley Medical College, Chennai, IND; 6 Medicine, Shifa Jeddah Polyclinic, Yanbu, SAU; 7 Medicine and Surgery, Shri Atal Bihari Vajpayee Medical College and Research Institute, Bengaluru, IND; 8 Surgery, Jonelta Foundation School of Medicine University of Perpetual Help System Dalta, Las Pinas, PHL; 9 Internal Medicine, Shaikh Khalifa Bin Zayed Al-Nahyan Medical and Dental College, Lahore, PAK; 10 Medicine, Universidad Católica de Santa María, Arequipa, PER; 11 Cardiology, Al-Quds Hospital, Gaza, PSE

**Keywords:** transcatheter aortic valve replacement, surgical aortic valve replacement, prosthetic valve endocarditis, one-year mortality, aortic valve replacement, aortic stenosis

## Abstract

Prosthetic valve endocarditis (PVE) is a rare but serious complication following aortic valve replacement using either a transcatheter aortic valve implantation (TAVI) or surgical aortic valve replacement (SAVR). This study aims to review the profiles and outcomes of PVE after surgical versus transcatheter aortic valve replacement. Electronic searches were performed on Scopus, EMBASE, and PubMed to retrieve related articles. To be included, study designs had to be randomized controlled trials (RCT) or observational cohort studies (in English) with PVE patients that compared differences based on TAVI or SAVR. This review included data for 13,221 patients with PVE diagnoses. Of those, 2,109 patients had an initial SAVR, and 11,112 patients had an initial TAVI. There was no difference in the incidence of PVE in patients who had initial TAVI versus SAVR (1.05% versus 1.01% per person-year, p=0.98). However, the onset of early PVE was more frequently observed in the TAVI group (risk ratio (RR): 1.54, 95% confidence interval (CI) [1.14, 2.08], p=0.005). Patients in the TAVI group had a lower indication for surgery to treat PVE when compared to SAVR (RR: 0.55, 95%CI [0.44, 0.69], p<0.001). *Staphylococcus aureus* was more likely to be the source of PVE in patients who had previous TAVI (RR: 1.34, 95%CI [1.17, 1.54], p<0.001). Also, *Enterococcus faecalis* was more frequently observed as a cause of PVE in the TAVI group (RR: 1.49, 95%CI [1.21, 1.82], p<0.001). Patients who underwent SAVR and TAVI had similar incidences of PVE. However, patients who underwent SAVR had a greater indication for surgery to treat PVE, while those who underwent TAVI had higher comorbidities, a higher likelihood of early PVE, and a trend towards higher one-year mortality.

## Introduction and background

Infective endocarditis (IE) is one of the most challenging diseases, requiring a multidisciplinary approach and attentive management. Prosthetic valve endocarditis (PVE) is a rare but serious complication following aortic valve replacement. Prosthetic valve IE has been estimated to account for 1% to 5% of all cases of IE [1]. The increasing number of surgical aortic valve replacements (SAVR) and transcatheter aortic valve implantation (TAVI) performed on patients with severe aortic stenosis annually leads to a higher incidence of PVE [2]. In cases of TAVI, paravalvular regurgitation and the space between the implanted prosthetic valve and the native valve can act as a nidus for IE [3,4]. Furthermore, a higher incidence of subclinical valve leaflet thrombosis in both SAVR and TAVI may also increase the risk for PVE [5]. The risk of postoperative PVE varies depending on the time elapsed since surgery, with a higher risk shortly after the procedure and a lower risk thereafter [6]. We performed a systematic review and meta-analysis to assess the comparative risk of prosthetic-specific IE after TAVI and SAVR. The main objectives of this systematic review were to evaluate and compare the incidence rates of infective endocarditis after TAVI and SAVR, PVE-associated short-term mortality, microbiological profile, one-year mortality, and the indication for surgery to treat PVE.

## Review

Method

Literature Search

Electronic searches were performed on Scopus, EMBASE, and PubMed from inception to October 2023. Search terms were formulated using the Population, Intervention, Comparator, and Outcome (PICO) framework to identify records comparing PVE following TAVI or SAVR. This study adhered to the updated 2020 version of the Preferred Reporting Items for Systematic Reviews and Meta-Analyses (PRISMA) guidelines [7]. The keywords employed in the search are as follows: ((Aortic Stenosis) OR (Aortic Valve Stenosis)) AND ((TAVI) OR (TAVR) OR (transcatheter aortic valve implantation)) AND ((SAVR) OR (surgical aortic valve replacement)) AND (infective endocarditis). A meticulous forward and backward citation check was conducted to ensure a comprehensive search and inclusion of all the relevant literature. The results were initially screened through the titles and abstracts of independent authors. Then, any conflicts were resolved by another author review. The final selection of the studies has satisfied the pre-established inclusion and exclusion criteria.

Studies Selection

**Figure 1:**
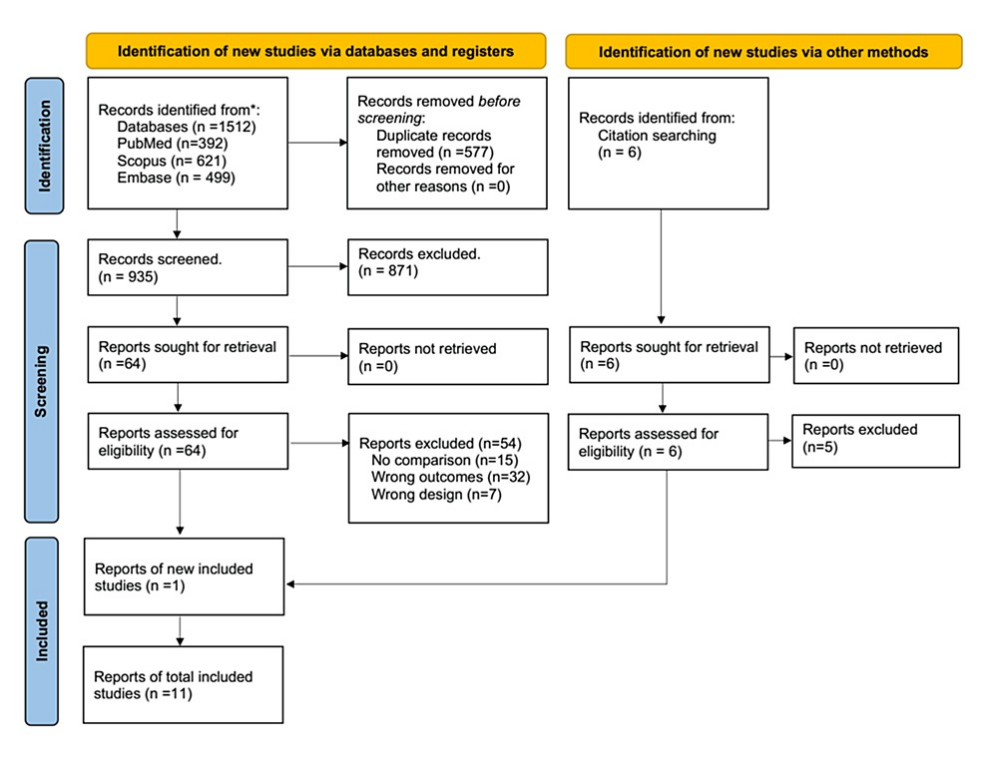
PRISMA flowchart detailing the process through which the final included articles were selected PRISMA: Preferred Reporting Items for Systematic Reviews and Meta-Analyses

To be included, study designs had to be randomized controlled trial (RCT) or observational cohort studies (in English) with at least five PVE patients that compared differences based on TAVI or SAVR and reported either one or all the following treatment outcomes: IE-related death, IE-embolization, indication for surgery, and PVE-microbiological profile (Figure 1). Studies with full-text availability were included. Excluded studies were systematic reviews, meta-analyses, narrative reviews, case reports/series, editorials, study protocols, abstracts, commentaries, and letters to the editor. Cohorts that included patients undergoing repeat SAVR, valve-in-valve TAVI, or repeat AVR for an indication other than IE were also excluded.

Data Extraction and Quality Assessment 

Two independent investigators were responsible for data extraction from each included article. Another two members further revised the obtained data, and any conflicts were resolved. Thus, consistency and accuracy were ensured.

The extracted data focused on study characteristics and key demographic data, including age, sex, and the occurrence of any comorbidities such as diabetes mellitus (DM), hypertension (HTN), chronic obstructive pulmonary disease (COPD), advanced heart failure, and chronic renal failure. The endpoint outcomes included in-hospital and one-year mortality, a microbiological profile, and indications for surgery to treat PVE. Categorical data were extracted as events and the total for each group, while continuous data were coded as mean and standard deviation. If the data were reported in other formats, the method by Wan et al. [8] was used to perform the necessary conversions. The main outcomes included incidence of IE, short-term mortality, which was defined as in-hospital or 30-day mortality, and one-year mortality. The secondary outcomes included indications for surgery and infectious etiology, including *Staphylococcus aureus, Enterococcus, *and* Streptococcus.*

Statistical Analysis

This meta-analysis was conducted per the guidelines outlined by the Cochrane Collaboration and the Meta-Analysis of Observational Studies in Epidemiology (MOOSE) [9]. Data analysis was performed using Review Manager Software version 5.4.1, a tool developed by the Cochrane Foundation. The Mantel-Haenszel random effects model was applied to calculate the risk ratio (RR) with the corresponding 95% confidence intervals (CI) for binary outcome measures. To evaluate the presence of statistical heterogeneity, we utilized the Q-test for heterogeneity (Cochrane, 1954) and I2 statistics. An I2 value exceeding 50% was considered indicative of substantial heterogeneity among the included studies. Statistical significance was defined by a p-value below 0.05. In order to ascertain the robustness of the findings, a sensitivity analysis was conducted. This analysis involved examining the impact of individual studies on the overall results.

Results

Characteristics of the Included Studies

Our literature search yielded a total of 1512 records from three unique databases. After removing 577 duplicates, 935 unique records were screened. Eventually, eleven articles that matched our eligibility criteria were included in this systematic review and meta-analysis. Our systematic review comprised ten studies, wherein patients were categorized into two groups based on their initial aortic valve replacement approach: TAVI or SAVR. The investigation aimed to analyze the incidence and profile of PVE. In total, our review encompassed data from 13,221 patients diagnosed with PVE. Among them, 11,112 patients initially underwent TAVI, and 2,109 patients underwent initial SAVR. Table 1 outlines the key characteristics of the studies included in our analysis. Table 2 summarizes the basic demographic data for the study population.

**Table 1:** Summary of the study characteristics y: years; m: months; d: days; SAVR: surgical aortic valve replacement; TAVI: transcatheter aortic valve implantation

S.No.	Study ID	Study duration	Study design	Number of patients		Total number of patients with IE		Follow-up period	
				SAVR	TAVI	SAVR	TAVI	SAVR	TAVI
1	Butt, 2019 [10]	2008-2016	Retrospective cohort	3777	2632	186	115	3.6 y	3.6 y
2	Cahill, 2022 [11]	2007-2016	Retrospective cohort	91962	14195	2057	140	53.9 m	24.5 m
3	Calderón-Parra, 2023 [12]	2015-2020	Prospective cohort	355	278	5	13	38 m (26-51)	41 m (24-56)
4	Fauchier, 2020 [13]	2010-2018	Retrospective cohort	60253	47553	2125	1127	2.0 y	1.2 y
5	Fernández-Avilés, 2022 [14]	2012-2020	Retrospective cohort	652	520	11	9	N/A	N/A
6	Kolte, 2018 [15]	2013-2014	Retrospective cohort	66077	29306	811	224	183 d (91-275)	153 d (91-275)
7	Lanz, 2021 [16]	2011-2018	Randomized trial	1828	2249	21	12	2.17 ± 1.51 y	2.17 ± 1.51 y
8	Shehada, 2018 [17]	2014-2015	Retrospective cohort	100	100	1	0	81 ± 6 m	69 ± 11 m
9	Summers, 2019 [18]		Randomized trial	1257	7273	12	95	2.69 ± 1.55 y	2.69 ± 1.55 y
10	Fukuhara, 2023 [19]	2011-2021	Retrospective cohort	N/A	N/A	5883	374	N/A	N/A
11	Panagides, 2024 [20]	2000-2020	Retrospective cohort	N/A	N/A	N/A	N/A	N/A	N/A

**Table 2:** Basic demographic data for the study population IE: infective endocarditis; Age and EuroScore are presented by mean ± standard deviation or median (interquartile range); DM: diabetes mellitus; TAVI: transcatheter aortic valve implantation; SAVR: surgical aortic valve replacement $ EuroSCORE was reported * STS-score **Charlson index

Study ID	Age		Sex (female)		% of bioprosthetic SAVR	Morbidity scores		Atrial fibrillation		DM	
	SAVR	TAVI	SAVR	TAVI		SAVR	TAVI	SAVR	TAVI	SAVR	TAVI
Butt, 2019 [10]	73 (68-78)	81 (77-85)	40.70%	47.60%	N/A	N/A	N/A	29.7%	38.9%	15.6%	18.4%
Cahill, 2022 [11]$	69.5 (59.5-76.6)	81.5 (75.8-85.0)	28.60%	29.30%	77.50%	6.19 (3.5-11.7)	18.82 (11.0-25.4)	14.6%	25.7%	13.7%	22.1%
Calderón-Parra, 2023 [12]**	77 (63-79)	75 (70-85)	0.0%	30.80%	N/A	5.7 ± 2.98	5.94±3.09	52.2%	53.6%	39.4%	40.5%
Fauchier, 2020 [13]**	71.96 ± 9.8	82.76 ± 6.7	36.50%	51.50%	N/A	4.1 ± 2.84	3.14 ± 2.8	N/A	N/A	N/A	N/A
Fernández-Avilés, 2022 [14]$	72 (70-79)	81 (78-82)	18.20%	44.40%	100%	3 (2-5)	6 (4-7)	N/A	N/A	N/A	N/A
Kolte,2018 [15]	67.0 ± 19.5	81.3 ± 12.2	38.60%	47.60%	N/A	N/A	N/A	36.7%	43.4%	27.3%	34.6%
Lanz, 2021 [16]*	76.5 ± 8.1	78.5 ± 5.6	33.30%	41.70%	100%	4.9 ± 1.9	4.9 ± 1.9	28.6%	25.0%	57.1%	58.3%
Shehada, 2018 [17]$	69±11	81±6	40%	42%	N/A	8.7±9.5	23.1±13.8	21%	42%	18%	39%
Summers, 2019 [18]	N/A	N/A	N/A	N/A	100%	N/A	N/A	N/A	N/A	N/A	N/A
Panagides, 2024 [20]	75.8 ± 7.6	76.3 ± 8.1	32.5%	31.0%	100%	N/A	N/A	N/A	N/A	27.0%	28.5%
Fukuhara, 2023 [19]*	63.0 (52.0-71.0)	73.0 (66.8-78.0)	20.10%	26.20%	65.50%	6.6 (4.1-10.9)	6.1 (3.6-10.7)	7.7%	15.2%	29.6%	44.1%

*Presentation of Prosthetic Valve Endocarditis* 

Across the majority of the included studies, patients who underwent initial TAVI were notably older [11,13,15,16] and presented with a higher burden of comorbidities, as evidenced by a higher Charlson Comorbidity Index (Charlson index 7 vs. 5, p=0.026) [16]. Additionally, Parra et al. [12] demonstrated that individuals who initially received TAVI were more prone to atypical presentations, characterized by an absence of fever (58.3% vs. 100%, p=0.086). Abscess formation or perivalvular extension was significantly higher in patients who underwent initial SAVR (8.3% vs. 47.6%, p=0.027) and (47.9% vs. 27%; P<.001), respectively [16,20]. Panagides et al. [20] showed in their matched cohort that patients who underwent initial SAVR presented with a higher incidence of moderate or severe new aortic regurgitation (43.4% vs. 13.5%; P<.001), whereas fewer cases of vegetation were identified in the SAVR group compared to the TAVR group (62.5% vs. 82%; P<.001).

Meta-Analysis Results

**Figure 2:**
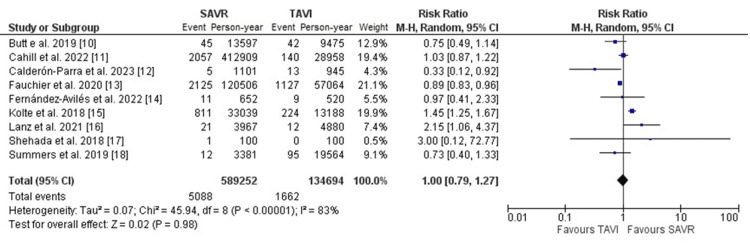
Forest plot illustrating the incidence of PVE per person per year subsequent to initial TAVI vs. SAVR TAVI: transcatheter aortic valve implantation; SAVR: surgical aortic valve replacement; PVE: prosthetic valve endocarditis [10-18]

The occurrence of PVE subsequent to the initial TAVI or SAVR demonstrated similar rates in both cohorts. In the TAVI group, the incidence stood at 1.05% per person-year, mirroring the 1.01% per person-year incidence observed in the SAVR group. The pairwise comparison revealed no significant difference between the two groups (RR: 1.00, 95% CI [0.79, 1.27], p=0.98) (Figure 2). However, it's imperative to acknowledge substantial heterogeneity across the encompassed studies. Early PVE was more frequently reported in patients who received TAVI (RR: 1.54, 95%CI [1.14, 2.08], p=0.005).

**Figure 3:**
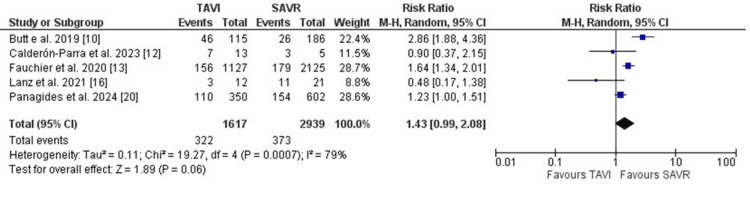
Forest plot illustrating the one-year mortality rates after PVE diagnosis PVE: prosthetic valve endocarditis [10,12,13,16,20]

**Figure 4:**
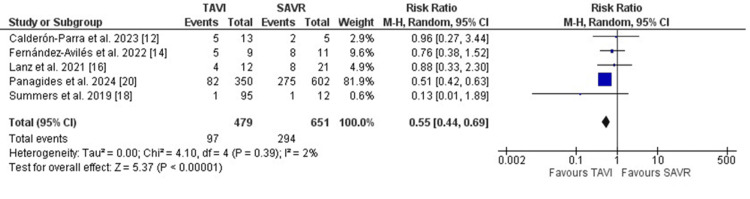
Forest plot illustrating the frequency of surgery indication to treat PVE between both groups PVE: prosthetic valve endocarditis [12,14,16,18,20]

Further, short-term mortality, including in-hospital or 30-day mortality, was comparable between both groups. (RR: 1.14, 95% CI [0.76, 1.69], p=0.53). Mortality rates after one year of follow-up were numerically higher in the TAVI group. However, the results did not reach statistical significance (RR: 1.43, 95% CI [0.99, 2.08], p=0.06) (Figure 3). The indication for surgery to treat PVE was less frequent in patients who initially received TAVI when compared to patients who underwent SAVR (RR: 0.55, 95%CI [0.44, 0.69], p<0.001) (Figure 4).

**Figure 5:**
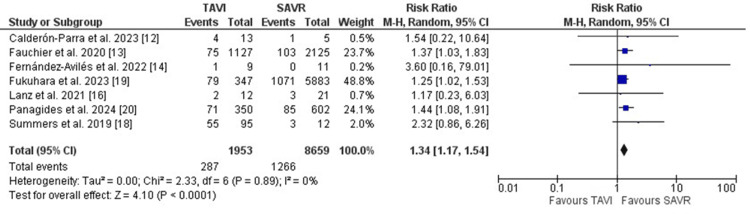
Forest plot illustrating the frequency of Staphylococcus aureus as the causative organism of PVE PVE: prosthetic valve endocarditis [12,13,14,16,18,19,20]

In terms of the microbiological profile, *Staphylococcus aureus, Streptococcus species, *and *Enterococcus faecalis* were the most frequently culture-identified organisms in PVE. PVE in patients who initially received TAVI was more likely to be caused by *Staphylococcus aureus* (RR: 1.34, 95%CI [1.17, 1.54], p<0.001) (Figure 5).

**Figure 6:**
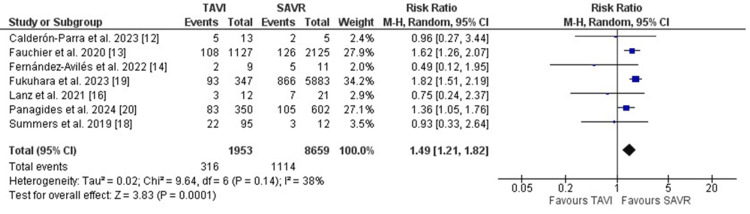
Forest plot illustrating the frequency of Enterococcus fecalis as the causative organism of PVE PVE: prosthetic valve endocarditis [12,13,14,16,18,19,20]

Similarly, *Enterococcus faecalis* was more frequently identified as the causative organism of PVE in patients who initially received TAVI (RR: 1.49, 95%CI [1.21, 1.82], p=0.0001) (Figure 6). On the other hand, the identification of *Streptococcus species* was not different between both groups (RR: 1.15, 95% CI [0.76, 1.73], p=0.50).

Discussion

Main Results of This Study

While there is still ongoing debate on whether TAVI should replace SAVR in patients suffering from severe aortic stenosis, there has been a growing interest in investigating the incidence and profile of PVE following these procedures. In this systematic review and meta-analysis, including data from ten studies, we aimed to investigate the differences in terms of outcomes and profile of IE in patients who have had TAVI or SAVR. The five main results of this study included: the incidence of PVE was not significantly different between patients who underwent TAVI or SAVR; short-term mortality after PVE was similar between both groups, and one-year mortality was numerically higher in the TAVI group; patients who initially underwent TAVI may present atypically, are older, and have higher comorbidities; *Staphylococcus aureus* and *Enterococcus faecalis* were more frequently identified as the cause of PVE in patients who initially underwent TAVI; there was no significant difference in the indication for surgery to treat IE between the two groups.

Discussion of the Pooled Results

Infective endocarditis signifies a severe infection linked to a range of serious complications and a poor prognosis. It can sometimes result in valve obstruction or malfunction, necessitating emergent surgical intervention [21,22]. The recent update of the modified Duke criteria by the International Society for Cardiovascular Infectious Diseases represents a significant advancement in the diagnosis and treatment of IE, broadening diagnostic capabilities and refining therapeutic approaches, thus underscoring the critical importance of addressing this disease [21]. Furthermore, severe complications such as congestive heart failure, perivalvular extension, and neurological involvement are associated with a poorer prognosis and increased early mortality rates [23-25].

Interestingly, our analysis revealed that the incidence of PVE did not significantly differ between patients who received TAVI and those who underwent SAVR. This finding suggests that both procedures may carry similar risks of PVE development, challenging the notion that TAVI might be associated with a higher incidence of PVE. Still, our analysis showed that the occurrence of “early” PVE was more frequently observed in the TAVI group. The space between the native valve and the implanted TAVI valve, the more frail TAVI population enrolled in most studies, and the increased permanent pacemaker requirement in TAVI patients are rational explanations for the increased incidence of early PVE among this group. Nonetheless, it is worth mentioning that patients in the SAVR group were frequently reported to have previous episodes of IE when compared to the TAVI group [20].

While short-term mortality rates were similar between PVE patients who initially underwent TAVI versus SAVR, the TAVI group had numerically higher one-year mortality rates with borderline significance (p=0.06). It is noteworthy to highlight that patients who underwent TAVI exhibit a higher surgical risk and more comorbidities [12,14]. Thus, the limited data on one-year mortality from only five studies may have affected the statistical power of drawing accurate conclusions. Although the complication rate is notably high, up to 90% of patients, it is important to note that there was no significant difference in PVE-associated complications between both groups (Alvis) [14].

Such increased mortality can be rationally explained by the more frail nature of the TAVI population included in most studies. Panagides et al. [25] define how the patients treated with TAVI have a different profile, as they have more comorbid and frail conditions than those undergoing SAVR, and also how some postprocedural complications are more frequently observed in patients with TAVR, such as the need for permanent pacemaker implantation, which may predispose to bacterial infections over time.

Results are still emerging regarding the PVE profile in patients with low surgical risk for whom TAVI was performed. The meta-analysis by Kolte et al. [26] showed a comparable incidence of IE after both procedures; however, they did not compare the detailed profile of IE in those patients.

The severity of IE following TAVI or SAVR is associated with patient profile, and it could be explained by several patient factors, including the presence of heart failure, stroke, persistent sepsis or bacteremia, septic emboli, vegetation, valvular dysfunction, or intracardiac abscess [27,28]. We realized how complex it is to distinguish between complications due to PVE, valve replacement, or other comorbidities. To emphasize further, Panagides et al. [25] showed that comorbidities such as chronic kidney disease can be an independent predictor of perivalvular extension in PVE in patients who initially underwent TAVI (adjusted odds ratio 2.08; 95% CI: [1.27-3.41]; p=0.003). Furthermore, PVE secondary to coagulase-negative staphylococci was also associated with an increased risk of local extension (adjusted odds ratio 2.71; 95% CI: [1.57-4.69]; p<0.001). Although the patients who undergo TAVI are at a higher risk in most of the published studies, we conclude that there is not enough evidence to establish any difference in the severity of PVE following TAVI as compared to SAVR.

Lastly, we analyzed the microorganisms most involved in the occurrence of infective endocarditis, with a high incidence of cases due to *Staphylococcus* and *Enterococcus* having a more prominent frequency in the TAVI group. Despite previous studies highlighting that the incidence of *Enterococcus spp.* endocarditis increases with age [29], we hypothesize it could be explained by the fact that most TAVI patients present with multiple comorbidities and thus require more in-hospital interventions such as catheterizations, which would increase the risk of bloodstream infection with *Enterococcus aureus*. Infection with *Enterococcus spp.* could be found even among patients with late IE after TAVI [30]. This might be due to the higher rate of comorbidity, such as poor bladder function, which might lead to an increased use of urinary catheterization or procedures involving the gastrointestinal system in the TAVI population, which could predispose to *Enterococcus spp.* infections leading to IE, as also suggested by Strange JE et al. [31]. Overall, several factors can play a role in causing this incidence.

Our analysis identified *Staphylococcus, Streptococcus*, and *Enterococcus* as the main microorganisms involved in PVE, with a higher frequency of *Staphylococcus* and *Enterococcus* observed in the TAVI group. The TAVI population typically presents with multiple comorbidities, necessitating frequent in-hospital interventions such as catheterizations. These interventions could be required for various reasons related to managing their comorbid conditions, such as urinary catheterization due to compromised bladder function or gastrointestinal procedures. These interventions increase the risk of bloodstream infections by *Enterococcus species*. Overall, the higher prevalence of comorbidities among TAVI patients contributes to the increased likelihood of hospital interventions, ultimately influencing the incidence of IE caused by microorganisms like *Enterococcus*.

Although years of experience with post-SAVR PVE have generated a considerable amount of evidence and several recommendations, this may not be the case for TAVI patients with PVE, who may present with different demographics, symptoms, and causative organisms. Hence, clinicians from different specialties should be familiar with the clinical presentation, diagnosis, and microbiology of TAVI-PVE in order to optimize the care of these patients and avoid detrimental complications.

Implications for Clinical Practice

Our study underscores the importance of close monitoring, patient education, multidisciplinary treatment approaches, and continued research to optimize the management of PVE following TAVI and SAVR.

 *Limitations and Future Directions*

Our study has several limitations. Firstly, it relies on aggregated study-level data, limiting granularity in analysis. Secondly, the majority of included studies were observational and retrospective, potentially introducing confounders and constraining the robustness of conclusions. Incorporating individual patient data and prospective designs into future research could enhance understanding of PVE outcomes post-TAVI and SAVR.

Moreover, the limited number of studies may have compromised the statistical power and generalizability. Multicenter studies with extended follow-up periods are needed to comprehensively assess the comparative outcomes of PVE following TAVI versus SAVR.

Furthermore, as TAVI expands to lower surgical-risk patients, investigating PVE incidence, profile, and outcomes in this population is crucial. Insights from such studies could inform tailored strategies for the prevention, diagnosis, and management of PVE in clinical practice.

## Conclusions

Our study highlights comparable incidence rates of PVE following TAVI and SAVR, with patients undergoing TAVI exhibiting higher comorbidities and a greater likelihood of early PVE occurrences. Although short-term mortality rates are similar, a trend towards higher one-year mortality is observed in the TAVI group. Notably, the SAVR group shows a greater indication for surgery to treat PVE.

## References

[1] Calderwood SB, Swinski LA, Waternaux CM, Karchmer AW, Buckley MJ (1985). Risk factors for the development of prosthetic valve endocarditis. Circulation.

[2] Conen A, Stortecky S, Moreillon P, Hannan MM, Franzeck FC, Jeger R, Widmer AF (2021). A review of recommendations for infective endocarditis prevention in patients undergoing transcatheter aortic valve implantation. EuroIntervention.

[3] Regueiro A, Linke A, Latib A (2016). Association between transcatheter aortic valve replacement and subsequent infective endocarditis and in-hospital death. JAMA.

[4] Del Val D, Panagides V, Mestres CA, Miró JM, Rodés-Cabau J (2023). Infective endocarditis after transcatheter aortic valve replacement: JACC state-of-the-art review. J Am Coll Cardiol.

[5] Chakravarty T, Søndergaard L, Friedman J (2017). Subclinical leaflet thrombosis in surgical and transcatheter bioprosthetic aortic valves: an observational study. Lancet.

[6] Ivert TS, Dismukes WE, Cobbs CG, Blackstone EH, Kirklin JW, Bergdahl LA (1984). Prosthetic valve endocarditis. Circulation.

[7] Page MJ, McKenzie JE, Bossuyt PM (2021). The PRISMA 2020 statement: an updated guideline for reporting systematic reviews. Syst Rev.

[8] Wan X, Wang W, Liu J, Tong T (2014). Estimating the sample mean and standard deviation from the sample size, median, range and/or interquartile range. BMC Med Res Methodol.

[9] Brooke BS, Schwartz TA, Pawlik TM (2021). MOOSE reporting guidelines for meta-analyses of observational studies. JAMA Surg.

[10] Butt JH, Ihlemann N, De Backer O (2019). Long-term risk of infective endocarditis after transcatheter aortic valve replacement. J Am Coll Cardiol.

[11] Cahill TJ, Raby J, Jewell PD (2022). Risk of infective endocarditis after surgical and transcatheter aortic valve replacement. Heart.

[12] Calderón-Parra J, de Villarreal-Soto JE, Oteo-Domínguez JF (2023). Risk of infective endocarditis associated with transcatheter aortic valve implantation versus surgical aortic valve replacement: a propensity score-based analysis. J Clin Med.

[13] Fauchier L, Bisson A, Herbert J (2020). Incidence and outcomes of infective endocarditis after transcatheter aortic valve implantation versus surgical aortic valve replacement. Clin Microbiol Infect.

[14] Fernández-Avilés C, Castillo JC, Heredia G, Resúa A, González R, Pan M, Anguita M (2022). Infective endocarditis on transcatheter aortic prosthesis: are there differences with endocarditis on surgically implanted aortic bioprosthesis?. Cardiol J.

[15] Kolte D, Goldsweig A, Kennedy KF (2018). Comparison of incidence, predictors, and outcomes of early infective endocarditis after transcatheter aortic valve implantation versus surgical aortic valve replacement in the United States. Am J Cardiol.

[16] Lanz J, Reardon MJ, Pilgrim T (2021). Incidence and outcomes of infective endocarditis after transcatheter or surgical aortic valve replacement. J Am Heart Assoc.

[17] Shehada SE, Wendt D, Peters D (2018). Infections after transcatheter versus surgical aortic valve replacement: mid-term results of 200 consecutive patients. J Thorac Dis.

[18] Summers MR, Leon MB, Smith CR (2019). Prosthetic valve endocarditis after TAVR and SAVR: insights from the PARTNER trials. Circulation.

[19] Fukuhara S, Wu X, Hawkins R, Ailawadi G, Deeb GM (2023). Prosthetic valve endocarditis after transcatheter and surgical aortic valve replacement. Ann Thorac Surg.

[20] Panagides V, Cuervo G, Llopis J (2024). Infective endocarditis after transcatheter versus surgical aortic valve replacement. Clin Infect Dis.

[21] Fowler VG, Durack DT, Selton-Suty C (2023). The 2023 Duke-International Society for Cardiovascular Infectious diseases criteria for infective endocarditis: updating the modified Duke criteria. Clin Infect Dis.

[22] Baddour LM, Wilson WR, Bayer AS (2015). Infective endocarditis in adults: diagnosis, antimicrobial therapy, and management of complications: a scientific statement for healthcare professionals from the American Heart Association. Circulation.

[23] Al-Tawil M, Friedrich C, Mandle K (2024). The influence of preoperative neurological complications on outcomes after surgery for infective endocarditis. Journal of Cardiac Surgery.

[24] Bohbot Y, Habib G, Laroche C (2022). Characteristics, management, and outcomes of patients with left-sided infective endocarditis complicated by heart failure: a substudy of the ESC-EORP EURO-ENDO (European infective endocarditis) registry. Eur J Heart Fail.

[25] Panagides V, Del Val D, Abdel-Wahab M (2022). Perivalvular extension of infective endocarditis after transcatheter aortic valve replacement. Clin Infect Dis.

[26] Kolte D, Vlahakes GJ, Palacios IF, Sakhuja R, Passeri JJ, Inglessis I, Elmariah S (2019). Transcatheter versus surgical aortic valve replacement in low-risk patients. J Am Coll Cardiol.

[27] Ivanovic B, Trifunovic D, Matic S, Petrovic J, Sacic D, Tadic M (2019). Prosthetic valve endocarditis - a trouble or a challenge?. J Cardiol.

[28] Pettersson GB, Hussain ST (2019). Current AATS guidelines on surgical treatment of infective endocarditis. Ann Cardiothorac Surg.

[29] Oliver L, Lavoute C, Giorgi R (2017). Infective endocarditis in octogenarians. Heart.

[30] Widmer D, Widmer AF, Jeger R, Dangel M, Stortecky S, Frei R, Conen A (2022). Prevalence of enterococcal groin colonization in patients undergoing cardiac interventions: challenging antimicrobial prophylaxis with cephalosporins in patients undergoing transcatheter aortic valve replacement. J Hosp Infect.

[31] Strange JE, Østergaard L, Køber L (2023). Patient characteristics, microbiology, and mortality of infective endocarditis after transcatheter aortic valve implantation. Clin Infect Dis.

